# Understanding and use of food labeling systems among Whites and Latinos in the United States and among Mexicans: Results from the International Food Policy Study, 2017

**DOI:** 10.1186/s12966-019-0842-1

**Published:** 2019-10-17

**Authors:** Claudia Nieto, Alejandra Jáuregui, Alejandra Contreras-Manzano, Edna Arillo-Santillan, Simón Barquera, Christine M. White, David Hammond, James F. Thrasher

**Affiliations:** 1 Nutrition and Health Research Center, Mexican National Institute of Public Health, Av. Universidad 655 Col, Santa María Ahuacatitlán, 62100 Cuernavaca, Mexico; 2 Population Health Research Center, Mexican National Institute of Public Health, Av. Universidad 655 Col. Santa María Ahuacatitlán, 62100 Cuernavaca, Mexico; 3 School of Public Health and Health Systems, University of Waterloo, 200 University Avenue West, Waterloo, ON N2L 3G1 Canada; 4 Department of Health Promotion, Education & Behavior, Arnold School of Public Health, University of South Carolina, 921 Assembly St, Columbia, SC 29208 USA; 5 School of Demography, ANU College of Arts and Social Sciences, The Australian National University, 9 Fellows Road Acton ACT 260, Canberra, Australia

**Keywords:** Food labelling, White, Latinos, Mexicans, Ethnicity

## Abstract

**Background:**

Obesity and chronic diseases could be prevented through improved diet. Most governments require at least one type of food labeling system on packaged foods to communicate nutrition information and promote healthy eating. This study evaluated adult consumer understanding and use of nutrition labeling systems in the US and Mexico, the most obese countries in the world.

**Methods:**

Adults from online consumer panels in the US (Whites *n* = 2959; Latinos *n* = 667) and in Mexico (*n* = 3533) were shown five food labeling systems: 1. Nutrition Facts Table (NFT) that shows nutrients of concern per serving; 2. Guideline Daily Amounts (GDA) that shows levels of nutrients of concern; 3. Multiple Traffic-Light (MTL) that color codes each GDA nutrient (green = healthy; yellow = moderately unhealthy; red = unhealthy); 4. Health Star Rating System (HSR) that rates foods on a single dimension of healthiness; 5. Warning Label (WL) with a stop sign for nutrients present in unhealthy levels. Participants rated each label on understanding (“easy”/“very easy to understand” vs “difficult”/“very difficult to understand”), and, for NFTs and GDAs, frequency of use (“sometimes”/“often” vs “never”). Mixed logistic models regressed understanding and frequency of use on indicators of labeling systems (NFT = ref), testing for interactions by ethnicity (US Latinos, US Whites, Mexicans), while controlling for sociodemographic and obesity-related factors.

**Results:**

Compared to the NFT, participants reported greater understanding of the WL (OR = 4.8; 95% CI = 4.4–5.3) and lower understanding of the HSR (OR = 0.34, 95% CI = 0.31–0.37) and the MTL (OR = 0.56, 95% CI = 0.52–0.61), with similar patterns across ethnic subgroups. Participants used GDAs less often than NFTs (OR = 0.48; 95%CI = 0.41–0.55), with the greatest difference among US Whites (OR = 0.10; 95%CI = 0.07–0.14).

**Conclusions:**

Understanding and use of the GDA was similar to that of the NFT. Whites, Latinos, and Mexicans consistently reported the best understanding for WLs, a FOPL that highlights unhealthfulness of a product. Therefore, a FOPL summary indicator, such as WLs, may be more effective in both the US and Mexico for guiding consumers towards informed food choices.

## Introduction

The highest rates of obesity in the world are in Mexico and the United States (US) [1, 2], where 72.5% [3] and 71.6% [4] of adults > 20 years old, respectively, are overweight or obese. Diabetes is also high in both countries at 9.4% [3, 5], along with other chronic diseases that could be prevented through improved diet. Most governments require at least one type of food labeling system on packaged foods to communicate nutrition information and promote healthy eating.

The National Health and Nutrition Examination Survey (NHANES) data has shown that 61.6% of Americans reported using a Nutrition Facts Table (NFT) [6], with a slightly lower percentage of use among US Latinos (60%) [7]. In contrast, only 41.5% of Mexicans reported reading the NFT [8]. In minority groups, like Latinos in the US, NFT comprehension is reported to be difficult compared to their white counterparts [9, 10].

The NFT is the oldest labeling system implemented on pre-packaged foods to inform consumers about nutrition content. The display of NFTs on packaged foods is mandatory in the US and Mexico, and is required by the National Labeling Act of 1990 and the Official Mexican Norm 051 (NOM-051), respectively. NFTs are displayed on the back or side of the package and provide information about the nutrition content per portion [8, 11, 12]. One limitation of NFTs is that serving sizes often differ across brands and products; studies suggest that consistent serving sizes would facilitate compare nutrient content of similar products [13]. Consumers also have difficulty using NFTs to understand if a food is ‘high’ or ‘low’ in a nutrient [14]. Perhaps most importantly, there are persistent disparities in the use and understanding of NFTs among consumers with lower education and income [15, 16].

To overcome limitations of NFTs, a number of Front-of-Package Labeling (FOPL) systems have been proposed and, in some countries, adopted to better communicate nutrition information to consumers [15, 17]. FOPLs provide summary information on energy and key nutrients, such as sugar, sodium, and saturated fat [18, 19], to facilitate healthy food choices [15, 17]. Many food-labeling systems have been proposed, including the FOPLs discussed below, yet there is no agreement on which is most effective.

**Fig. 1:**
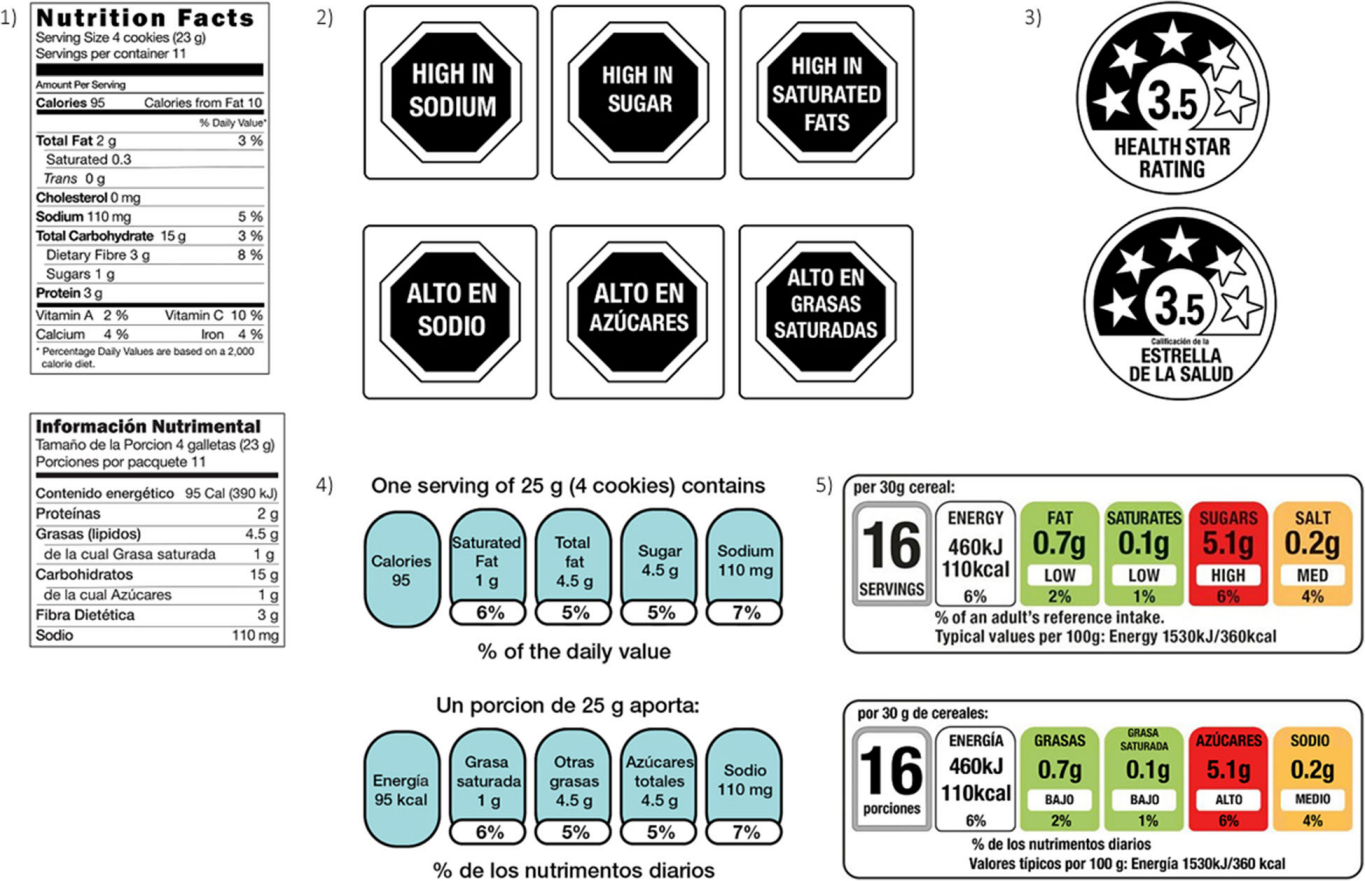
Food labelling systems evaluated. 1) Nutrition Facts Table, 2) Warning Labels, 3) Health Star Rating, 4) Guideline Daily Amounts, and 5) Multiple Traffic Light

Warning Labels (WL) are displayed on the front of packages for energy and nutrients considered to be present in unhealthy levels (see Fig. 1). Products with WLs are perceived as less healthful than those with numerical FOPL systems [20, 21]. The World Health Organization has recognized that interpretive FOPL, like WLs, can help create healthier food environments because they are more easily understood by consumers at all levels of literacy and also indirectly motivate companies to put healthier products on the market [22]. Furthermore, the Pan American Health Organization indicated that WLs should be used as a tool in the design and implementation of various regulatory strategies related to the prevention and control of overweight and obesity [23]. Chile, Israel, Peru, and Uruguay recently adopted WLs as a nutrition policy tool; WLs are also under consideration in Brazil, Canada, and Mexico.

One FOPL approach favored by industry is the Guideline Daily Amounts (GDA), which shows energy and the percentage of key nutrients of concern (saturated fat, other fat, sugar and sodium) on the front of the package (see Fig. 1). GDAs have been mandatory on the front of packaged foods in Mexico since 2014. In the US, industry voluntarily uses a similar system called Facts Up Front [24], for many products. US consumers report better understanding of nutrition information presented in the GDA format than in NFTs [25]. However, US consumers show evidence of greater nutrition knowledge when using other systems (e.g., traffic light) compared to the GDA [26]. Furthermore, Mexicans show evidence of low understanding of GDA formatted information [8, 27].

The Health Star Rating (HSR) synthesizes all nutrient information into a single dimension of healthiness. The HSR was voluntarily implemented by the food industry in 2014 in Australia and New Zealand. Products receive ratings from half a star up to 5 stars, depending on the overall healthiness of the product [28]. Studies suggest that the HSR can be more effective at directing consumers towards healthier choices compared to the GDA [29].

Finally, the Multiple Traffic Light (MTL) color codes each nutrient in order to quickly communicate whether the product contains relatively low (green), average (yellow) or high (red) levels of potentially harmful nutrients [20]. Consumers using the MTL have been shown to have more accurate reports of calories per serving compared to the GDA, HSR and the single traffic light [30]. Additionally, MTLs have high acceptability among Europeans [31, 32].

This study assessed labeling systems currently used by the food industry in the US and Mexico, as well as other systems adopted by other countries. The objective was to compare adult consumers’ understanding and use of five food labeling systems (NFT, WL, GDA, HSR, and MTL; see Fig. 1). Our approach is oriented by the framework of consumer decision-making proposed by Grunert, in which awareness is a necessary precursor to understanding food labels. Once labeling is understood, it may influence food choices [33]. We compared US Whites, US Latinos, and Mexicans, partly because Latinos are the largest minority group in the US [34] and the majority are of Mexican heritage [35]. Furthermore, US Latinos have disproportionately high rates of obesity and lower health literacy than Whites [36–38]. Furthermore, comparisons with Mexican consumers allowed assessment of mandated GDAs in Mexico relative to the voluntary use of FOPLs in the US.

## Methods

We analyzed cross-sectional survey data from the US and Mexico administrations of the 2017 International Food Policy Study. The sample included 7159 participants aged 18 to 64 who completed an online survey in December 2017. Participants were recruited through the Nielsen Consumer Insights Global Panel and their partners. In US, participants self-reported their ethnicity. We used data from participants in the US who considered themselves ‘White’ or ‘Hispanic or Latino’; and all participants in Mexico. The Nielsen panels were originally recruited using both probability and non-probability sampling methods in each country. For the current project, Nielsen targeted recruitment, so that the percentage of participants in each age group would be similar to the general population for each country. Latino respondents were over-sampled in the US (*n* = 667), to facilitate comparisons between US Whites (*n* = 2959) and Mexicans (*n* = 3533). Respondents provided consent prior to completing the survey. Participants received remuneration in accordance with their panel’s usual incentive structure (e.g., points-based or monetary rewards, chances to win prizes). Post-stratification weights were constructed separately for each country to weight the sample so that it was similar to known sex, age, and region distributions in the general population. Surveys were conducted in Spanish in Mexico, and US participants could choose to answer either an English- or Spanish-language survey. Native and bilingual Spanish speakers on the research team reviewed the Spanish translations independently. The average time to complete the survey in US and Mexico was 35.7 min. The study was reviewed by and received ethics clearance through a University of Waterloo Research Ethics Committee (ORE# 21460). A full description of the study methods can be found in the International Food Policy Study: Technical Report – Wave 1 (2017) at www.foodpolicystudy.com/methods.

### Food labeling systems evaluated

Participants were asked to evaluate five different food labeling systems: 1) NFT, 2) WL, 3) GDA, 4) HSR, and 5) MTL (Fig. 1). Participants were shown an image of each labelling system, one at a time, and asked to answer two questions about each label before proceeding to the next label: 1) how easy or difficult the information was to understand (responses re-coded to ‘easy/very easy to understand’ vs ‘neither easy nor difficult’ or ‘difficult/very difficult to understand’), and 2) how often they used that type of label when choosing food to purchase (responses recoded to ‘often/sometimes’ vs ‘never’). The label images were shown on screen with the question (one question and image per screen). Label images were presented isolated from other images rather than being part of a food package. The researchers did not give any explanation to interpret or assess the labeling systems shown.

### Covariates

Covariates included socio-demographic characteristics and other variables relevant to food choices, including sex (male or female), age group (18 to 33, 34 to 49, or 50 to 64 years old), education (high school or lower, technical studies, or bachelor’s degree or higher) and survey language (English or Spanish). Income adequacy was assessed with the question ‘Thinking about your total monthly income, how difficult or easy is it for you to make ends meet?,’ with responses collapsed into difficult, neither easy nor difficult, or easy. Occupation was classified as ‘full-time worker or full-time student’, ‘part-time worker and/or part-time student’ or ‘unemployed’.

Body Mass Index (BMI) measurement followed World Health Organization criteria [39], wherein self-reported height and weight were used to classify participants as underweight (18.5 kg/m^2^), normal weight (18.5 to 24.9 kg/m^2^), overweight (25.0 to 29.9 kg/m^2^), or obese (> 30 kg/m^2^). Self-reported nutrition knowledge was assessed with the question ‘How would you rate your nutrition knowledge?’ with responses collapsed into not knowledgeable, or somewhat knowledgeable / knowledgeable. Daily calorie count was queried by asking ‘Do you count calories you consume each day?’ with responses including ‘never’, ‘sometimes’, and ‘most of the time’. Household responsibility for food shopping was assessed by asking whether the participant did most of the food shopping in their household (‘yes’, ‘no’, or ‘share equally with others’).

### Statistical analysis

Pearson chi square tests were used to evaluate sample differences by ethnicity (US Whites, US Latinos, and Mexicans). Prevalence and 95% confidence intervals were estimated for understanding and use of each food labeling system, both overall and by ethnicity.

Crude and adjusted mixed-effects logistic models were estimated by regressing understanding of food labels (0 = difficult/very difficult/neither easy nor difficult, 1 = easy/very easy) on each type of labeling system (NFT = Reference group). This approach was also used in models where label use (0 = never, 1 = often/sometimes) was the outcome and labeling systems currently in use in both countries were the independent variable (i.e., GDA and NFT) (NFT = Reference group). Adjusted models included all covariates described in methods. Since our initial models indicated that significant differences in label understanding existed across ethnicities, models were estimated both for the entire sample and stratified by ethnicity. We used the F-test to test for significance (*p* < 0.05). Model fit was tested with goodness of fit of the model. All analysis were performed in STATA, version 14 (StataCorp, L.P., College Station, TX).

## Results

A total of 7159 adults were included in the present study. US participants who were neither White nor Latino were excluded from the analysis (*n* = 118). Participants with missing data for the covariates and dependent variables were also excluded from the analysis (*n* = 1345). In the total sample (*n* = 7159), 39% [95% CI: 38.36–41.09] were US whites, 10% [95% CI: 9.46–11.22] were US Latinos, and 50% [95% CI: 48.57–51.35] were Mexicans (Table 1). Across ethnicities, US white participants had the highest proportion of older adults (41.8%, [95%CI: 39.64–43.94]), self-reported obesity (26.4%, [95% CI: 24.54–28.42]), participants reporting it was easy to make the ends meet (53.3%, [95% CI: 51.02–55.46]) and being a full-time worker or student (61.1%, [95% CI: 58.92–63.22]) (*p* < 0.001 for all). Mexican participants had higher education level (bachelor’s degree or higher) (71.8%, [95% CI: 70.08–73.50]), reported that they were somewhat knowledgeable about nutrition (55.1%, [95% CI: 53.21–57.05]) and reported that they never counted calories (70.8%, [95% CI: 69.06–72.58]) (*p* < 0.05 for all). A higher proportion of US Latino participants reported doing most of the food shopping for their household (75.3%, [95% CI: 71.17–79.07]) (*p* < 0.05). The majority of US Latinos (67.4%, [95% CI: 63.14–71.46]) answered the survey in Spanish.

**Table 1:** Socio-demographic characteristics of White, Latino and Mexican population who participated in the IFPS (*n* = 7159)*

	White (*n* = 2959)	Latino (*n* = 667)	Mexican (n = 3533)	*P* value^a^
	% [95% CI]	% [95% CI]	% [95% CI]	
Sex				
Female	49.88 [47.65–52.10]	49.51 [44.99–54.03]	51.42 [49.49–53.33]	*p* = 0.5313
Male	50.12 [47.89–52.34]	50.49 [45.96–55.00]	48.58 [46.66–50.50]	
Age group				
18–33	28.62 [26.74–30.57]	42.15 [37.82–46.59]	42.74 [40.92–44.57]	*p* < 0.001
34–49	29.60 [27.41–31.88]	45.96 [41.42–50.55]	35.53 [33.72–37.38]	
50–64	41.78 [39.64–43.94]	11.89 [9.46–14.85]	21.72 [19.82–23.74]	
Survey language				
English	100	32.55 [28.53–36.85]	0	*p* < 0.001
Spanish	0	67.45 [63.14–71.46]	100	
Education level				
High school or lower	18.48 [16.79–20.30]	24.34 [20.73–28.34]	15.68 [14.39–17.05]	*p* < 0.001
Technical studies	20.02 [18.28–21.87]	20.61 [17.07–24.65]	12.49 [11.24–13.85]	
Bachelor’s degree or higher	61.50 [59.29–63.64]	55.05 [50.50–59.51]	71.83 [70.08–73.50]	
Income adequacy				
Difficult	16.46 [14.86–18.18]	29.84 [25.80–34.21]	41.55 [39.64–43.47]	*p* < 0.001
Neither easy nor difficult	30.29 [28.28–32.37]	36.87 [32.59–41.36]	38.32 [36.46–40.20]	
Easy	53.25 [51.02–55.46]	33.29 [29.23–37.60]	20.14 [18.66–21.69]	
Occupation				
Worker or student	61.10 [58.92–63.22]	54.18 [49.65–58.63]	55.50 [53.57–57.40]	*p* < 0.001
Unemployed	24.45 [22.63–26.35]	20.31 [17.02–24.04]	14.41 [13.08–15.85]	
Part time student and/or pt-worker	14.45 [12.95–16.08]	25.51 [21.75–29.66]	30.09 [28.37–31.86]	
BMI^b^				
Normal weight	38.02 [35.89–40.18]	39.43 [35.10–43.93]	44.72 [42.82–46.64]	*p* < 0.001
Overweight	35.54 [33.40–37.73]	37.38 [33.10–41.85]	36.27 [34.41–38.15]	
Obesity	26.44 [24.54–28.42]	23.19 [19.54–27.27]	19.01 [17.53–20.57]	
Self-report of nutrition knowledge				
Not knowledgeable	26.87 [24.89–28.94]	27.41 [23.57–31.60]	29.92 [28.22–31.67]	*p* < 0.001
Somewhat knowledgeable	43.45 [41.25–45.65]	51.44 [46.91–55.95]	55.14 [53.21–57.05]	
Knowledgeable	29.68 [27.71–31.73]	21.15 [17.65–25.11]	14.94 [13.52–16.47]	
Daily calorie count				
Never	62.03 [59.85–64.16]	56.57 [52.03–60.99]	70.86 [69.06–72.58]	*p* < 0.001
Sometimes	31.16 [29.14–33.24]	36.61 [32.35–41.08]	25.57 [23.93–27.28]	
Most of the time	6.81 [5.76–8.02]	6.82 [4.92–9.37]	3.57 [2.85–4.44]	
Food shopping in your household				
Yes	66.44 [64.26–68.55]	75.34 [71.17–79.07]	67.97 [66.19–69.68]	*p* < 0.001
No	10.94 [9.45–12.61]	8.16 [5.94–11.11]	8.66 [7.71–9.70]	
Share	22.62 [20.83–24.50]	16.49 [13.37–20.16]	23.38 [21.83–24.99]	

95% CI = 95% Confidence Intervals

*Data were weighted using survey weights

^a^Pearson χ2 tests were calculated to determine differences by socio-demographic characteristics and ethnicity

^b^Body Mass Index (BMI) classification: normal weight (18.5 to 24.9 kg/m2), overweight (25.0 to 29.9 kg/m2), and obesity (> 30 kg/m2)

**Fig. 2:**
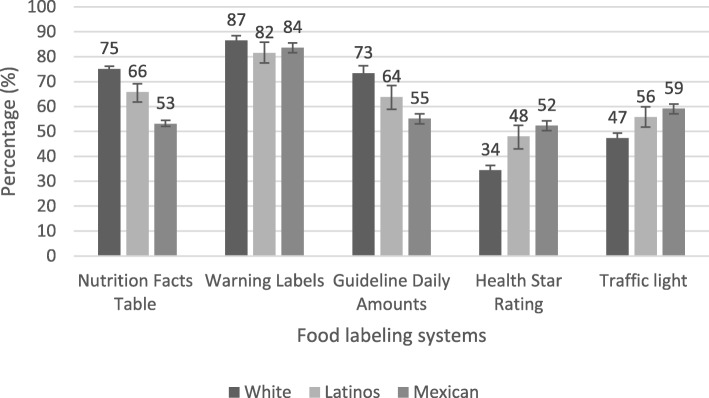
Understanding (easy/very easy) of food labelling systems

Figure 2 presents the prevalence of reported understanding of food labeling systems among Whites, Latinos, and Mexicans. Across ethnicities, Whites reported the highest level of understanding (87, 95% CI: 85.91–88) for WL, and reported lowest understanding of the HSR (34, 95% CI: 32.30–36.63) and the MTL (47, 95% CI: 45.10–49.57) labels. Latinos and Mexicans also reported high understanding for WL (82 and 84%, respectively).

**Fig. 3:**
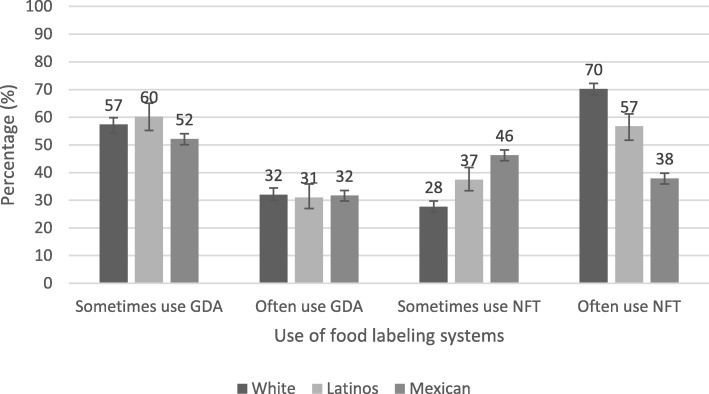
Use (sometimes/often) of the Guideline Daily Amounts and Nutrition Facts Table

Figure 3 shows the use of the GDA and the NFT. US Latinos reported the highest levels of use for the GDA and NFT labeling systems (GDA: 91, 95% CI: 88–94; NFT: 94, 95% CI: 92–96%). While 31% reported using GDAs “sometimes” and 60% reported using them “often”. Almost all Whites reported using the NFT (98, 95% CI: 97–98%): 28% reported using the NFT “sometimes”, while 70% reported using the NTF “often”. A lower percentage of Mexicans reported using GDAs (84, 95% CI: 82–85) and NFTs (84, 95% CI: 83–86%).

Across the total sample, the adjusted model for label understanding (Table 2) indicated that participants were 4.82 (95%CI: 4.39–5.30) times more likely to report understanding the WL compared to the NFT. In contrast, reported understanding of the HSR (OR = 0.34, 95% CI: 0.31–0.37) and the MTL (OR = 0.56, 95% CI: 0.52–0.61) were lower compared to the NFT.

**Table 2:** Odds ratios for considering it easy or very easy to understand different food labelling systems*

	Total sample (*N* = 35,618)		White (*n* = 14,692)		Latino (*n* = 3316)		Mexican (*n* = 17,610)	
	%	OR [95% CI]	%	OR [95% CI]	%	OR [95% CI]	%	OR [95% CI]
Labelling Scheme (Ref. NFT)								
Warning Labels	85	4.82 [4.39–5.30] ***	87	2.90 [2.49–3.38]***	82	4.07 [2.92–5.68]***	84	7.95 [6.89–9.16]***
Guideline Daily Amounts	63	1.02 [0.95–1.09]	73	0.94 [0.83–1.07	64	1.04 [0.80–1.37]	55	1.08 [0.98–1.19]
Health Stars Rating	45	0.34 [0.31–0.37]***	34	0.09 [0.08–0.11]***	48	0.35 [0.25–0.47]***	52	0.92 [0.81–1.04]
Multiple Traffic Light	54	0.56 [0.52–0.61]***	47	0.19 [0.17–0.22]***	56	0.52 [0.39–0.69]***	59	1.31 [1.17–1.45]***
Ethnicity (Ref. Latino)								
White	63	0.89 [0.75–1.05]	–	–	–	–	–	–
Mexican	61	0.96 [0.82–1.13]	–	–	–	–	–	–
Sex (Ref. Man)								
Woman	63	1.10 [1.01–1.20]*	65	1.27 [1.11–1.46]***	65	1.34 [0.93–1.93]	61	0.97 [0.86–1.11]
Age (years)		1.00 [1.00–1.00]		1.00 [1.00–1.01]		1.00 [0.98–1.01]		1.00 [0.99–1.00]
Survey language (Ref. English)								
Spanish	56	1.02 [0.75–1.38]	–	–	67	1.18 [0.83–1.66]	–	–
Education (Ref. High-school or lower)								
Technical studies	64	1.08 [0.93–1.25]	65	0.97 [0.78–1.22]	68	1.71 [1.01–2.90]*	61	1.07 [0.85–1.36]
Bachelor’s degree or higher	62	0.91 [0.81–1.02]	63	0.85 [0.71–1.03]	63	1.08 [0.71–1.64]	61	0.93 [0.78–1.11]
Income adequacy (Ref. Difficult)								
Neither easy nor difficult	60	1.04 [0.94–1.16]	60	0.82 [0.68–1.00]*	60	0.89 [0.60–1.34]	61	1.15 [1.00–1.31]*
Easy	66	1.40 [1.25–1.56]***	65	1.18 [0.98–1.41]	65	1.32 [0.87–2.00]	67	1.53 [1.28–1.82]***
Occupation (Ref. Unemployed)								
Part time worker or student	62	0.92 [0.82–1.03]	63	0.88 [0.75–1.04]	56	0.74 [0.47–1.14]	62	0.97 [0.80–1.18]
Worker or student	62	0.96 [0.87–1.07]	64	0.93 [0.77–1.11]	66	0.52 [0.34–0.79]***	61	1.08 [0.94–1.25]
BMI (Ref. Normal weight)								
Overweight	62	0.92 [0.83–1.01]	64	1.00 [0.86–1.16]	59	0.74 [0.50–1.10]	60	0.92 [0.80–1.06]
Obesity	61	0.94 [0.84–1.05]	64	1.05 [0.89–1.24]	64	1.11 [0.72–1.71]	58	0.83 [0.70–0.98]*
Nutrition Knowledge (Ref. Not knowledgeable)								
Somewhat Knowledgeable	62	1.41 [1.28–1.56]***	62	1.23[1.05–1.43]**	62	1.70 [1.15–2.51]**	62	1.51 [1.31–1.75]***
Knowledgeable	69	1.97 [1.75–2.22]***	73	1.76 [1.48–2.08]***	68	3.19 [1.99–5.14]***	73	2.04 [1.66–2.51]***
Calorie count (Ref. Never)								
Sometimes	66	1.28 [1.17–1.41]***	67	1.30 [1.13–1.50]***	59	0.75 [0.52–1.09]	67	1.40 [1.21–1.62]***
Most of the time	69	1.51 [1.25–1.81]***	69	1.64 [1.28–2.10]***	73	1.14 [0.60–2.18]	68	1.49 [1.06–2.10]*
Food shopping (Ref. Yes)								
No	59	0.90 [0.78–1.04]	64	1.03 [0.83–1.29]	62	0.84 [0.44–1.58]	54	0.82 [0.66–1.01]
Share	62	1.03 [0.93–1.14]	64	1.15 [0.99–1.34]	63	0.99 [0.65–1.50]	60	0.97 [0.84–1.13]

OR = Odds Ratio; 95% CI = 95% Confidence Intervals

Mixed models were adjusted for sex, age, survey language, education, income adequacy, occupation, BMI, nutrition knowledge, calorie count, and food shopping in household

Significance levels: *P* value * < 0.05, ** < 0.01,*** < 0.001

*Data were weighted using survey weights

The understanding of FOPL systems relative to NFT differed by ethnicity (*p* < 0.05 for the interaction term Ethnicity x labelling system, data not shown). Compared to Latinos, Mexicans were more likely to report understanding the WL (OR = 2.15, 95% CI 1.54–3.01), HSR (OR = 2.44, 95% CI: 1.80–3.32) and MTL (OR = 2.39, 95% CI = 1.79–3.17) than the NFT. Compared to Latinos, Whites had lower odds for understanding the HSR and MTL (HSR OR = 0.23, 95% CI = 0.17–0.32.; MTL OR = 0.34, 95% CI = 0.25–0.46).

When stratified by ethnicity (Table 2), participants were more likely to report understanding the WL compared to the NFT (*p* < 0.05) across all ethnicities (Whites OR = 2.90, 95% CI = 2.49–3.38; Latinos OR = 4.07, 95% CI = 2.92–5.68; Mexican OR = 7.95, 95% CI = 6.89–9.16), and all ethnicities were less likely to report understanding the GDAs compared to the NFT (*p* < 0.05). The MTL and HSR had a lower rating of understanding among Whites (MTL = 0.19, 95% CI: 0.17–0.22; HSR = 0.09, 95% CI: 0.08–0.11) and Latinos (MTL = 0.52, 95% CI: 0.39–0.69; HSR = 0.35, 95% CI: 0.25–0.47), whereas the MTL had a better rating of understanding among Mexicans (MTL = 1.31, 95% CI: 1.17–1. 45), compared to the NFT.

Adjusted models of label use for the full sample indicated that participants were less likely (OR = 0.48, 95% CI = 0.41–0.55) to report using GDAs compared to the NFT (Table 3). In the full sample, women, participants with a higher level of education, more nutrition knowledge, reporting to find it easy to make ends meet, and those who reported to count calories had a higher odds of using food labels. The use of labels differed by ethnicity (*p* < 0.05 for the interaction term Ethnicity x labelling system).

**Table 3:** Odds ratios for often or sometimes using the Guideline Daily Amounts*

	Total sample (*N* = 19,534)		White (*n* = 6848)		Latino (*n* = 2009)		Mexican (*n* = 10,686)	
	%	OR [95% CI]	%	OR [95% CI]	%	OR [95% CI]	%	OR [95% CI]
Labelling Scheme (Ref. NFT)								
Guideline Daily Amounts	86	0.48 [0.41–0.55]***	89	0.10 [0.07–0.14]***	91	0.53 [0.32–0.88]**	84	0.93 [0.78–1.11]
Ethnicity (Ref. Latino)								
White	90	0.80 [0.50–1.28]	–	–	–	–	–	–
Mexican	82	0.33 [0.22–0.51]***	–	–	–	–	–	–
Sex (Ref. Man)								
Woman	87	1.28 [1.06–1.54]***	92	1.59 [1.19–2.13]***	90	0.84 [0.47–1.50]	83	1.28 [0.99–1.66]
Age (years)		1.01 [1.00–1.02]**		1.01 [1.00–1.02]		1.01 [0.98–1.03]		1.00 [0.99–1.01]
Survey language (Ref. English)								
Spanish	56	1.32 [0.73–2.39]	–	–	67	1.52 [0.86–2.69]	–	–
Education (Ref. High-school or lower)								
Technical studies	85	1.36 [1.01–1.84]*	91	1.53 [0.97–2.40]	90	1.43 [0.70–2.94]	78	1.28 [0.83–1.98]
Bachelor’s degree or higher	87	1.64 [1.30–2.08]***	91	1.51 [1.03–2.21]*	93	1.30 [0.70–2.41]	84	1.90 [1.35–2.66]***
Income adequacy (Ref. Difficult)								
Neither easy nor difficult	85	1.28 [1.03–1.59]	90	0.99 [0.67–1.47]	90	1.46 [0.78–2.73]	82	1.23 [0.93–1.62]
Easy	90	1.42 [1.12–1.80]**	91	1.10 [0.75–1.61]	92	1.31 [0.69–2.47]	87	1.43 [1.01–2.02]*
Occupation (Ref. Unemployed)								
Part time worker or student	86	0.95 [0.75–1.22]	90	1.22 [0.85–1.76]	92	0.27 [0.14–0.52]***	83	0.94 [0.64–1.38]
Worker or student	86	1.03 [0.83–1.28]	90	0.93 [0.63–1.35]	92	0.71 [0.38–1.34]	82	1.05 [0.79–1.40]
BMI (Ref. Normal weight)								
Overweight	86	0.86 [0.70–1.06]	89	0.75 [0.55–1.03	92	1.51 [0.79–2.88	83	0.91 [0.69–1.21]
Obesity	85	0.84 [0.66–1.06]	91	1.06 [0.74–1.51]	90	1.25 [0.63–2.45	79	0.66 [0.47–0.93]*
Nutrition Knowledge (Ref. Not knowledgeable)								
Somewhat Knowledgeable	87	2.43 [1.99–2.97]***	91	1.64 [1.18–2.29]**	90	1.64 [0.90–2.98]	91	3.26 [2.49–4.28]***
Knowledgeable	93	3.45 [2.63–4.52]***	93	1.91 [1.32–2.76]***	92	2.33 [1.10–4.91]*	93	7.29 [4.57–11.61]***
Calorie count (Ref. Never)								
Sometimes	94	5.49 [4.40–6.85]***	93	2.33 [1.72–3.15]***	95	3.95 [2.09–7.49]***	94	11.73 [8.25–16.68]***
Most of the time	95	4.67 [2.90–7.54]***	96	3.42 [1.84–6.35]***	93	3.16 [1.14–8.81]*	94	6.62 [3.02–14.49]***
Food shopping (Ref. Yes)								
No	80	0.56 [0.41–0.75]**	89	0.96 [0.61–1.49]	84	0.68 [0.27–1.72]	73	0.38 [0.25–0.59]***
Share	84	0.77 [0.62–0.96]	89	0.97 [0.70–1.34]	89	0.75 [0.37–1.53]	80	0.73 [0.55–0.97]*

*OR* Odds Ratio, 95% *CI* 95% Confidence Intervals

Mixed models were adjusted for sex, age, survey language, education, income adequacy, occupation, *BMI*, nutrition knowledge, calorie count, and food shopping in household

Significance levels: *P* value * < 0.05, ** < 0.01,*** < 0.001

*Data were weighted using survey weights

When stratified by ethnicity, the use of the GDA remained significant for Whites (OR = 0.10, 95% CI = 0.07, 0.14) and Latinos (OR = 0.53, 95% CI = 0.32–0.88) only. Both ethnicities were less likely to report using the GDAs compared to the NFT. In all stratified models, women, participants with a higher level of education, more nutrition knowledge, reporting to find it easy to make ends meet, and those who reported to count calories had a higher odds of using food labels.

## Discussion

Our study indicated that understanding and use of the GDA is similar to that of the NFT, suggesting that GDAs may not provide additional guidance to consumers to make informed food choices. Even in Mexico, where implementation of the GDA on the front of the packages was accompanied with a massive media campaign sponsored by the food industry, this labeling scheme was not more understood or used than the NFT. This is not surprising given that GDAs communicate the same nutrient numbers displayed in NFTs, with relatively little interpretative information compared to other FOPL systems. Our results also suggest differences in the understanding and use of food labeling schemes across ethnicities.

The GDA is currently displayed on the packages of processed foods in both Mexico and US [24, 40]. This non-interpretative labeling format was introduced by the food industry as their response to the need to provide simplified nutrition information for consumers [41]. However, studies in Mexico and other countries indicated that the GDA may not help consumers to make informed food purchases and does not promote healthy food choices due to difficulties understanding quantitative nutrient amounts, even among highly educated populations [8, 27, 42]. In line with these findings, in our study the self-reported understanding and use of the GDA was similar to NFTs across the three populations. Like NFTs, the GDA requires consumers to do mathematical calculations which may be a determining factor for a poor understanding [43]. Indeed, consumers demonstrate even greater deficits in understanding when consumers are asked to apply nutrient numbers featured in GDAs in functional tests, rather than simply self-report their level of understanding [20, 44]. Additionally, the nutritional criteria used for estimating GDA are not based on international benchmarks [43]. Taken together, these findings support the growing evidence suggesting that GDA is an inadequate FOPL to promote healthy food choices among consumers.

In our study we also tested the understanding of other semi-directive (HSR and MTL) labeling formats, which in theory have better potential to guide consumers towards healthy food choices [45, 46]. Interestingly, the reported understanding for the HSR was lower than that of the NFT among Whites and Latinos, whereas among Mexicans, the understanding of this labeling format was similar to the NFT. However, a qualitative study among Hispanic adults showed that front of package labels with star formats were not easily understood or liked [12]. Additionally, since consumers in the US and Mexico are not familiar with the HSR, this may contribute to their reported difficulty understanding the HSR.

Ecuador successfully implemented the MTL which helped consumers reduce the consumption of products with high levels of fat, sugar and salt [47]. In the present study, the MTL was also associated with lower reported understanding compared to the NFT among Latinos and Whites, but not Mexicans. This is in line with a study showing good subjective understanding for the MTL among Mexican consumers [12]. Differences in the understanding of labels by ethnicities also support the hypothesis that labels should be targeted towards specific populations [48], as cultural factors may determine the effectiveness of a label [7, 9]. Additionally, different acculturation levels between Whites and Latinos may influence the use of nutrition information [10, 49].

WLs appeared to be the most effective FOPL format for Mexicans, Latinos and Whites. Despite some differences in understanding across ethnicities, this label format consistently had the highest rating for reported understanding. The high ratings for WLs might be explained by the finding that interpretive labels that include information on product unhealthfulness tend to better support consumers to choose nutritionally favorable products [22]. Our findings are consistent with previous studies in Latin American adults showing that WLs improve consumer’s ability to correctly identify products containing excessive amounts of critical nutrients, compared with the GDA system [20, 50]. Studies conducted in Brazil and Uruguay demonstrated the ease and rapidity of WL understanding at the point of sale [20, 50]. For example, in a randomized experiment in Brazil, WLs improved understanding of excess nutrient content (27.0% versus 8.2%, *p* < 0.001); and helped participants to correctly identify healthier products (14.0% versus 6.9%, *p* < 0.001) [50]. A study in Uruguay found that introducing WLs reduced response time when shopping compared to the GDA [20].

Overall, women had higher odds of understanding the labels than men. In prior studies, females tend to better understand and use food labels [48, 51] compared to men. Other research among Latino women in the US indicates that they are less acculturated than Latino men [37, 52], nevertheless in our Latino sample we did not find statistically significant differences by sex. We integrated a proxy measure of acculturation into our analyses by adjusting for language of survey administration; however, acculturation is a complex process and enriched measures may be needed to better understand whether lower acculturation among Latina women helps explain our counterintuitive findings [53, 54]. Our results show that Latinos with higher education levels have higher odds of understanding food labels; other international studies have also found similar results [55–59]. Whites reported a higher labeling understanding compared to Latinos. On the other hand, Latinos reported a higher use of labels. This might be explained by the Latino participant’s desire to please the interviewer; some studies have documented that Latinos have a higher probability of acquiescence response bias [60].

### Strengths and limitations of the study

This is the first food labeling study that compares White, Latino and Mexican populations, and as such may be of international interest to several countries who are looking to modify their current food labelling policies. This study is subject to a variety of limitations. First, the use of an online survey might have biased the findings in Mexico as 40% of the Mexican population does not have internet access and internet is often restricted to certain residential areas [61]. Also, the Mexico sample had higher levels of education [3], likely resulting in higher levels of understanding and use of labeling than would be found in the general population. A similar pattern was observed for education among US Whites and Latinos [62], but the education level was particularly high in the Mexico sample. However, the analysis was adjusted by education in statistical models when examining differences across the ethnic sub-groups. BMI was somewhat lower in our sample than for national estimates. This may be partially explained by the known under-reporting for weight and over-reporting for height [63, 64]; however, it may also mean that our sample had healthier food patterns and therefore may have been more likely to use labels than the general population.

Additionally, the present study assessed self-reported use of labels, which is likely to over-estimate actual use [31, 65]. Label understanding was also self-reported, although subjective understanding provides a reasonable approximation on the extent to which consumers believe they have “understood” what is being communicated, and this likely reflects the effectiveness of the label [31]. Future research should use protocols that involve a more objective assessment of understanding with real food products and behavioral outcomes, such as purchasing. Finally, US Latino participants did not provide information about their specific heritage and there may be important differences in our outcomes across specific ethnic subgroups of Latinos. Still, it is likely that most of our Latino sample was of Mexican heritage, given that almost two-thirds of US Latinos are from this ethnic subgroup [34].

## Conclusions

Our study found that understanding and use of the GDA was similar to that of the NFT, suggesting that this labeling format may not provide much additional guidance to consumers to make healthier food choices. Whites, Latinos, and Mexican participants consistently reported the best understanding when using WLs that highlight the unhealthfulness of a product. A FOP summary indicator, such as WLs, may be effective in both the US and Mexico for guiding consumers towards informed food choices.

## Data Availability

The datasets used and/or analyzed during the current study are available from the corresponding author on reasonable request.

## Abbreviations

BMI: Body Mass Index
FOPL: Front-of-Pack Labeling
GDA: Guideline Daily Amounts
HSR: Health Star Rating
MTL: Multiple Traffic Light
NFT: Nutrition Facts Table
US: United States
WL: Warning Label
